# Balance of IL-10 and Interferon-γ plasma levels in human visceral leishmaniasis: Implications in the pathogenesis

**DOI:** 10.1186/1471-2334-5-113

**Published:** 2005-12-19

**Authors:** Arlene Caldas, Cecília Favali, Dorlene Aquino, Vera Vinhas, Johan van Weyenbergh, Cláudia Brodskyn, Jackson Costa, Manoel Barral-Netto, Aldina Barral

**Affiliations:** 1 Centro de Pesquisas Gonçalo Moniz, Fundação Oswaldo Cruz – FIOCRUZ, Salvador, BA, Brazil; 2 Department of Nursing, Federal University of Maranhão, UFMA, São Luís, MA, Brazil; 3 Faculdade de Medicina da Bahia. Universidade Federal da Bahia, UFBA, Salvador, BA, Brazil; 4 Institute of Investigation in Immunology, Salvador, Bahia, Brazil

## Abstract

**Background:**

Leishmaniasis remains a serious public health problem in several parts of the developing world. Effective prophylactic measurements are hampered by imprecise comprehension of different aspects of the disease, including its immunoregulation. A better comprehension of immunoregulation in human VL may be useful both for designing and evaluating immunoprophylaxis.

**Methods:**

To explore immunoregulatory mechanisms, 20 visceral leishmaniasis (VL) patients were evaluated during active disease and at different periods up to one year after treatment determining their plasma cytokine levels, clinical parameters (palpable spleen and liver) and antibody levels.

**Results:**

Elevated plasma levels of IFN-γ and of IL-12 p40 were observed during active disease, significantly decreasing after treatment whereas in vitro Leishmania antigen-stimulated IFN-γ production by PBMC exhibited an inverse pattern being low during disease and increasing steadily thereafter. Absence of IFN-γ activity is a hallmark of VL. The main candidate for blunting IFN-γ activity is IL-10, a cytokine highly elevated in plasma with sharp decrease after treatment. Activity of IL-10 is inferred by high levels of anti-Leishmania specific IgG1 and IgG3. TGF-β had elevated total, but not of active, levels lessening the likelihood of being the IFN-γ counterpart. Spleen or liver size presented a steady decrease but return to normal values at only 120 days after treatment. Anti-Leishmania IgG (total and subclasses) levels and DTH or Leishmania-stimulated lymphocyte proliferation conversion to positive also present a slow decrease after treatment. IL-6 plasma levels were elevated in only a few patients.

**Conclusion:**

Taken together our results suggest that IFN-γ and IL-10 are the molecules most likely involved in determining fate of disease. After treatment, there is a long delay before the immune profile returns to normal what precludes using plasma cytokine levels as criteria of cure as simpler clinical evaluations, as a palpable spleen or liver, can be used.

## Background

Leishmaniasis remains a serious public health problem in several parts of the developing world. Effective prophylactic measurements are hampered by imprecise comprehension of different aspects of the disease, including its immunoregulation. A better comprehension of immunoregulation in human VL may be useful both for designing and evaluating immunoprophylaxis.

Leishmaniases present a wide spectrum of manifestations, with either tegumentary or visceral involvement. Visceral leishmaniasis (VL) is a progressive infection with fatal outcome in the absence of treatment. During disease evolution there is extensive parasite multiplication attaining a high parasite burden in the spleen and liver. Whereas in the most common forms of tegumentary leishmaniasis parasite growth is controlled and an anti-leishmania cell-mediated immune response (CMI) is mounted [1], lack of anti-leishmania CMI has been considered a hallmark of VL [2,3] There has been described a transient incapacity of mounting an effective CMI in VL based on the absence of lymphocyte proliferative response as well as IL-2 or IFN-γ production upon PBMC in vitro stimulation by leishmanial antigen [2-4]. VL patients also have negative delayed-type hypersensitivity skin tests. [5]

The existence of antigen-reactive T cells was suspected since both in vitro and vivo CMI evaluations turn positive following cure [2,3,6,7]. Even during active disease, anti-leishmania IgG3 and IgG4 antibody is detected suggesting an active T-cell control of antibody production [8]. Additionally, there is evidence of IFN-γ production during active human VL as shown by the presence of mRNA in spleen samples [9] and the presence of high levels of IFN-γ, and/or of IFN-γ inducing cytokines in plasma [10-13]. Albeit present, IFN-γ is not capable of fully exerting its activities probably due to the presence of counter regulatory products such as IL-10 [14,15].

In the present report, we took advantage of studying a group of VL patients during active disease and performing a long follow-up in order to determine their plasma cytokine levels. Evaluating the presence of cytokines during active VL and at various time periods after effective treatment maps the recovery of the cytokine pattern and helps understanding immunoregulatory mechanisms in this complex disease.

## Methods

### Study design

A prospective study was conducted on 20 patients with VL, including 12 patients seen at clinics from the Brazilian public health system and eight patients seen at the Infectious and Parasitic Diseases outpatient clinic at the Federal University of Maranhão (UFMA), São Luís, MA, Brazil. Patients (10 males and 10 females) participated in this study. Clinical and demographic characteristics of the patients are given in Table I.

**Table 1:** Clinical and hematological data on 20 patients with visceral leishmaniasis.

Patient	Age	Spleen			Liver			Weight			Erythrocytes			Hemoglobin		
	years	Size (cm)		% var*	Size (cm)		% var*	kg		% var*	mm3		% var*	g/dl		% var*
		d0	d30		D0	D30		D0	D30		D0	D30		D0	D30	
01	5	5	0**	-1.00	4	0	-1.00	15	16.5	0.10	2.6	4.3	0.65	7.6	10.5	0.38
02	6	5	4	-0.20	10	8	-0.20	18.5	19.0	0.03	2.7	4.2	0.56	8.0	10.6	0.31
03	1	7	4.5	-0.36	5.5	4	-0.28	7.4	7.9	0.07	1.8	3.5	0.94	3.5	7.9	1.26
04	1	8	4	-0.50	7	4	-0.43	8.0	8.2	0.03	4.1	4.6	0.12	7.6	8.5	0.12
05	3	6.5	3	-0.53	6.5	4	-0.38	11.8	11.9	0.01	2.9	3.0	0.03	5.2	5.4	0.04
06	2.5	7	3	-0.57	7	5	-0.29	11.4	11.5	0.01	3.2	3.8	0.19	8.4	10.9	0.30
07	2	6	3	-0.50	6	4	-0.33	9.6	9.6	0.00	2.5	3.7	0.48	4.0	7.0	0.75
08	9	7	0	-1.00	8.5	6	-0.29	22.5	24.2	0.07	3.0	3.6	0.20	7.8	9.6	0.23
09	5	8.5	3.5	-0.59	7.5	6	-0.20	15.0	15.5	0.03	3.4	3.7	0.09	6.6	7.0	0.06
10	2	8	3	-0.63	8	6	-0.25	11.8	13.0	0.11	3.91	4.8	0.23	8.9	10.6	0.18
11	5	4	3	-0.25	5	3	-0.40	18.0	19.0	0.06	3.0	5.3	0.77	9.3	11.0	0.18
12	6	4	0	-1.00	8	6	-0.25	15.0	15.5	0.03	3.7	4.0	0.08	9.0	10.4	0.16
13	1	6	3	-0.50	7	5	-0.29	6.0	8.3	0.38	2.5	3.4	0.36	7.3	9.8	0.34
14	5	3	0	-1.00	6	3	-0.50	14.9	15.3	0.03	2.5	4.6	0.84	7.3	12.2	0.67
15	16	20	17.5	-0.13	7	5	-0.29	45.0	48.0	0.07	3.0	4.8	0.60	9.0	11.6	0.28
16	23	12	8	-0.33	8	6	-0.25	60.0	65.9	0.10	2.5	3.8	0.52	6.5	8.8	0.35
17	33	6.5	4.5	-0.54	4.5	3	-0.33	39.0	40.0	0.03	3.0	3.3	0.10	8.1	9.2	0.14
18	29	8	4	-0.50	6	4	-0.33	59.0	61.9	0.05	2.8	3.8	0.36	8.4	11.4	0.36
19	51	4.5	0	-1.00	10	3	-0,70	56.6	58.1	0.03	3.9	3.0	-0.23	8.9	8.0	-0.10
20	18	14	5	-0.64	0	0	0.00	52.6	53.3	-0.06	3.4	4.5	0.32	7.3	11.0	0.51

Mean	11.2	7.5	3.8	-0.59	6.6	4.25	-0.35	25.06	26.13	0.06	3.02	3.99	0.36	7.43	9.55	0.33
SD	13.4	3.9	3.9	0.28	2.22	1.97	0.20	19.47	20.09	0.09	0.59	0.63	0.30	1.61	1.80	0.30

* % variation = (d30-d0)/d0.

**0 = not palpable;

The study was performed between August 2000 and July 2002. The study was approved by the Research Ethics Committee of "Hospital Universitário Presidente Dutra", and all individuals included in this report, or their legal guardians, signed an informed consent form before enrollment. Patients living in the municipalities of São Luís Island (São Luís, São José de Ribamar, Paço do Lumiar and Raposa), who had not started VL treatment, and who agreed to participate in the study were included in the study. Patients who did not adhere to regular follow-up defined as attendance of the scheduled return visits were excluded. Diagnosis was confirmed by identification of amastigote forms of *Leishmania sp *in Giemsa-stained smears of bone marrow aspirates. All patients received N-methylglucamine antimonate (Glucantime^®^) as the drug of first choice at the dose of 20 mg/Sb^5+^/kg/day (maximum dose of 810 mg/day), intravenously, over a period of 20 to 30 days. Two adult patients complained of myalgia at the beginning of treatment but discontinuation of the drug was not required. No patient in this series exhibited marked anemia (3+/4+ and/or hemoglobin ≤ 5 g/dl), hemorrhagic phenomena, severe diarrhea or severely impaired general and/or nutritional status. Eight individuals with associated respiratory infection were treated in hospitals. No differences in clinical or laboratory parameters were observed between hospitalized patients and those treated in outpatient clinics.

Data were collected using a standard protocol chart containing the following information: identification, clinical complaints, physical exam, and the results of laboratory tests performed before (day zero) and after therapy (30 days). All patients underwent clinical follow-up every 10 days during treatment, monthly up to 3 months after treatment, and then every 3 months until completing 12 months. Liver and spleen sizes were evaluated by two observers by palpation and reported as the distance to the rib border in cm. Since the significance of the reduction of a palpable liver or spleen depends on the patient size, such results are presented both as reduction in cm and as "percent variation" calculated subtracting the post treatment (30 days) value from the initial value and then dividing the result by the initial value. Clinical assessment consisted of recording the symptoms reported by the patient or responsible person and complete clinical examination including measurement of spleen and liver size and weight.

A group of normal volunteers (19) was included in our study. They were individuals from the same endemic area, with the same range age and presenting negative anti-*Leishmania *serology and negative anti-parasite DTH.

### Samples

Peripheral blood (maximum 20 ml) was collected for PBMC and plasma. The blood was diluted 1:1 in culture medium, layered on Ficoll-Hypaque (Sigma-Aldrich, St Louis, MO) and centrifuged at 400 g for 30 min at room temperature PBMC recovered from the interface, were washed twice by centrifuging at 300 g for 10 min.

### Antigen

Soluble *Leishmania *antigen (SLA) was prepared as described elsewhere [2] Briefly, stationary phase promastigotes of *Leishmania chagasi *were ultrasonicated and centrifuged at 20,000 × g for 2 h. The supernatant was used at a final concentration of 10 μg/ml.

### Cell culture

PBMC were obtained from heparinized venous blood by passage over a Ficoll Hypaque gradient (Sigma-Aldrich). PBMC were washed three times and resuspended at a concentration of 5 × 10^6 ^cells /ml in RPMI 1640 medium (Gibco, NY) supplemented with 2 mM L- glutamine, penicilin (100 U/ml), streptomicin (100 μg/ml) (Gibco, NY) and 10% heat inactivated human AB serum (Sigma-Aldrich). Cells were plated in 24 well tissue culture plates (Costar, Corning Incorporated, NY) at a concentration of 5 × 10^6 ^cells/ml and incubated at 37°C, 5% CO_2_. Stimulation was performed by addition of SLA (10 μg/ml). PBMC were stimulated with anti-CD28 plus anti-CD3 (10 μg/ml) as positive controls. Supernatants were harvested 24, 48 and 96 hours after *in vitro *stimulation and maintained at -20°C until use.

### Proliferation assays

For lymphoproliferation assays, PBMC (1 × 10^6 ^cells/ml) were plated in 96 well flat bottom plates, 200 μl/well and stimulated with SLA or anti-CD28 and anti-CD3 as a positive control, for 5 days at 37°C and 5% CO_2_. Lymphoproliferation was accessed by evaluation of [3H]thymidine incorporation in beta counter after a 8 hour pulse of cell cultures with [3H]thymidine (1 μCi/well). Incorporation of radioactive label was measured by liquid scintillation (Cell Harvester, FilterMate 196 Perkin Elmer Life Sciences, MA). Results are expressed as the mean counts per minute in triplicate cultures (Direct Beta Counter Matrix 9600, PerkinElmer Life Sciences, MA)

### Cytokine ELISA

IL-10, IFN-γ, IL-6 (B&D, San Diego, CA) and TGF-β (Promega, Madison, WI) levels were determined on culture supernatants and plasma using commercially available ELISA kits. Cultures supernatants were harvested at different time points: for IFN-γ we harvested the supernatant at 72 hours and for IL-10 48 hours after the addition of stimulus (SLA antigen at 10 μg/ml) We measured active and total TGF-β, treating the samples by acidification or not, respectively. The assays were performed according to the manufacturer's instructions.

### Delayed-type hypersensitivity reaction

DTH was performed as previously described [2], using *L chagasi *promastigotes (MHOMBR-83-BA-3) antigen. The test was applied intradermally to the anterior forearm, and readings were taken 48 to 72 hours after application (CUBA *et al*.,1985; WHO,1990), with the diameter of the induration measured with a millimeter ruler. One or both diameters equal to or greater than 5 mm were considered to be a positive reaction. The test was carried out on all patients before treatment, at the end of treatment and each month afterwards up to DTH conversion.

### ELISA serology

Antileishmanial antibodies were detected by ELISA. Soluble leishmanial antigen (SLA) from *L. chagasi *was diluted in buffer (15 mM Na2HCO3, 28 mM NaH CO3, pH 9.6) to 1 ug/well (50 ul) to coat 96-wells microtiter plates (Probind; Falcon, Becton Dickinson, Lincoln Park, NJ) by overnight incubation at 4°C. A solution of PBS plus 1% Tween 20 was used for one hour at room temperature to decrease the background. After five washes with PBS plus 0.1% Tween 20 (PBS-T), sera diluted at 1:100 was added at 50 μl/well and incubated 30 minutes at room temperature. After another washing cycle, a 1:10,000 dilution of alkaline phosphatase-conjugated anti-human IgG antibody (Sigma Immunochemicals, St. Louis, MO) was incubated at room temperature for 30 minutes. For IgG sub-classes reactivity, the incubation with human sera (1:100 dilution) was followed by one hour incubation with a 1:1000 solution of anti-human IgG1, IgG2, IgG3 or IgG4 monoclonal antibodies (Sigma Immunochemical). After further washing, plates were treated for one hour with a 1:10000 dilution of alkaline phosphatase-conjugated anti-mouse Ig (Sigma Immunochemical). After further washings, plates were developed using a chromogenic solution of p-nitrophenylphosphate in sodium carbonate buffer pH9.6 with 1 mg/ml of MgCl_2_. The absorbance was recorded at 405 nm.

### Statistical analysis

Data were analyzed by both between subjects (unpaired cases) and within subject (paired cases) using Kruskal-Wallis test followed by Dunn's post test with GraphPad software version 4.0 (GraphPad Software Inc, San Diego, CA, USA)

## Results

### Clinical parameters

**Figure 1:**
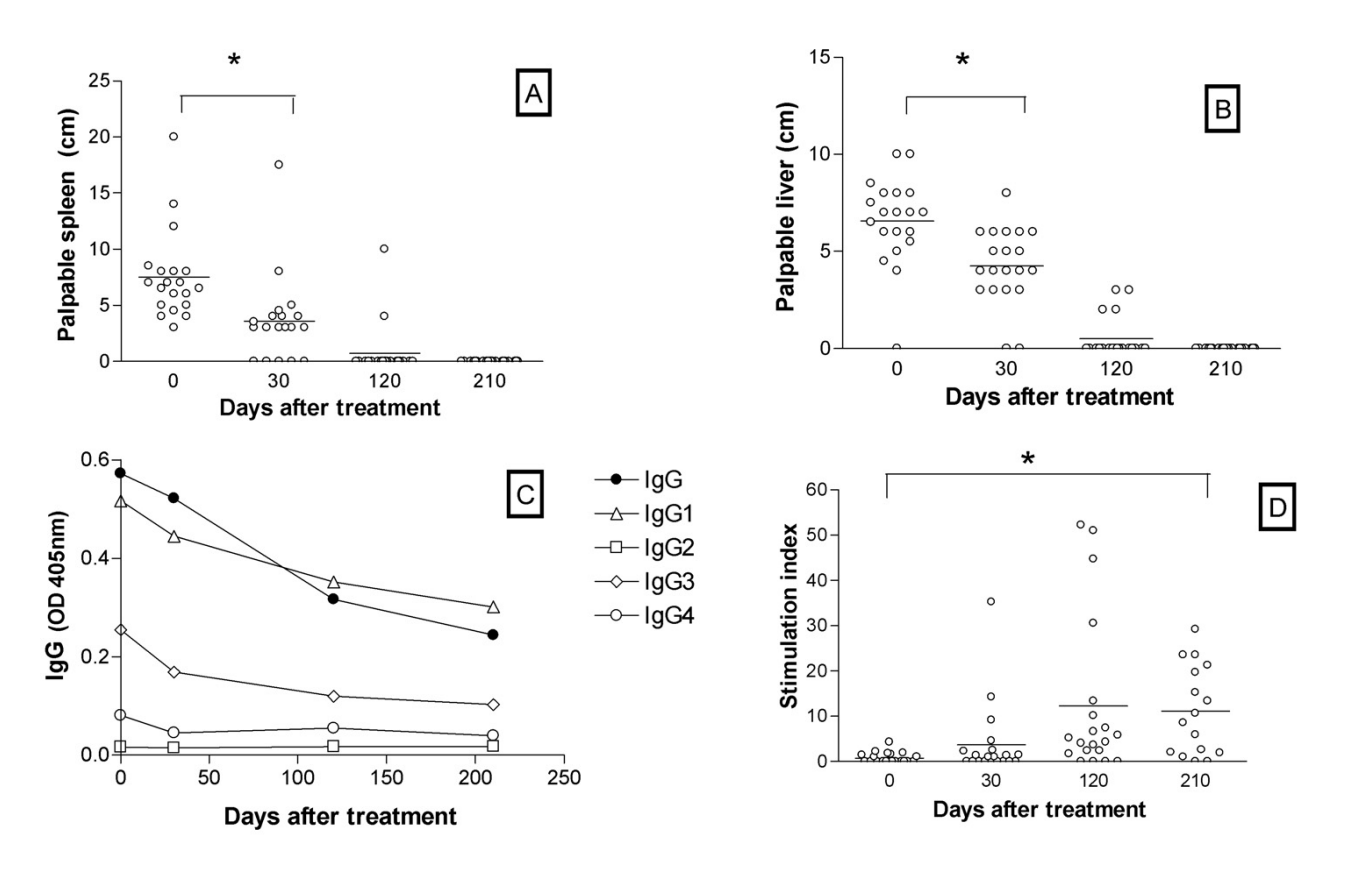
**Clinical and immunological parameters from acute VL at different periods after treatment. **Spleen (A) liver (B) size and plasma IgG levels and their subclasses (C) from VL patients during acute VL (day 0) and at different periods after treatment (30, 120 and 210 days) are shown. Stimulation index obtained in proliferation assay from PBMC from VL stimulated with Leishmania antigen (10 μg/ml) are represented in D.

Reduction of enlarged spleen or liver with return to normal impalpable stage has long been used to evaluate cure in human VL. In the present series reductions of spleen (Fig. 1A) or liver (Fig. 1B) were observed 30 days after treatment (p < 0.0001 in both cases), but only few patients had normal values then. Impalpable organs were observed in almost all patients only 120 days after treatment.

### Antibody levels and cell mediated immune responses

Production of different subclasses of IgG can be used as a surrogate marker of cellular immune responses developed by the patients. With this perspective,, we evaluated anti-leishmania total IgG levels, as well as its subclasses, during active VL and at different times after treatment. Anti-leishmania antibodies are present in all subclasses except for IgG2, with a predominance of IgG1 and a modest increase of IgG4 (Fig. 1C). All elevated IgG levels drop gradually, but only IgG4 returned to below cut-off levels 30 days after treatment. Evaluation of anti-leishmania IgE did not exhibit a decreasing pattern after treatment (data not shown).

Conversion of DTH to positive demonstrates the recovery of CMI in VL patients. However, our results show that conversion of DTH was positive in less than 50% of tested individuals 210 days after treatment, reaching 100% conversion only after 390 days of cure (data not shown). Despite being submitted to repeated DTH reactions the patients in the present series exhibited a slow rate of DTH conversion, a finding against a possible sensitization by the antigen. Leishmania antigen-stimulated PBMC proliferation has also been largely used for gauging the appearance of anti-parasite specific cell-mediated immunity in VL patients. Only two patients of the present series had stimulation indexes above two during active disease (Fig. 1D), with these numbers increasing to only seven by 30 days after treatment and to 15 positive tests at 120 days post-treatment (Fig. 1D). Increase of stimulation index was significant (p = 0.0014) but differences between each time point and day 0 were significant only at day 210 post-treatment. We used anti-CD3 and anti-CD28 as positive controls in these proliferation assays and we verified that the stimulation indexes ranged from 20 to 30.

### Cytokine levels

**Figure 2:**
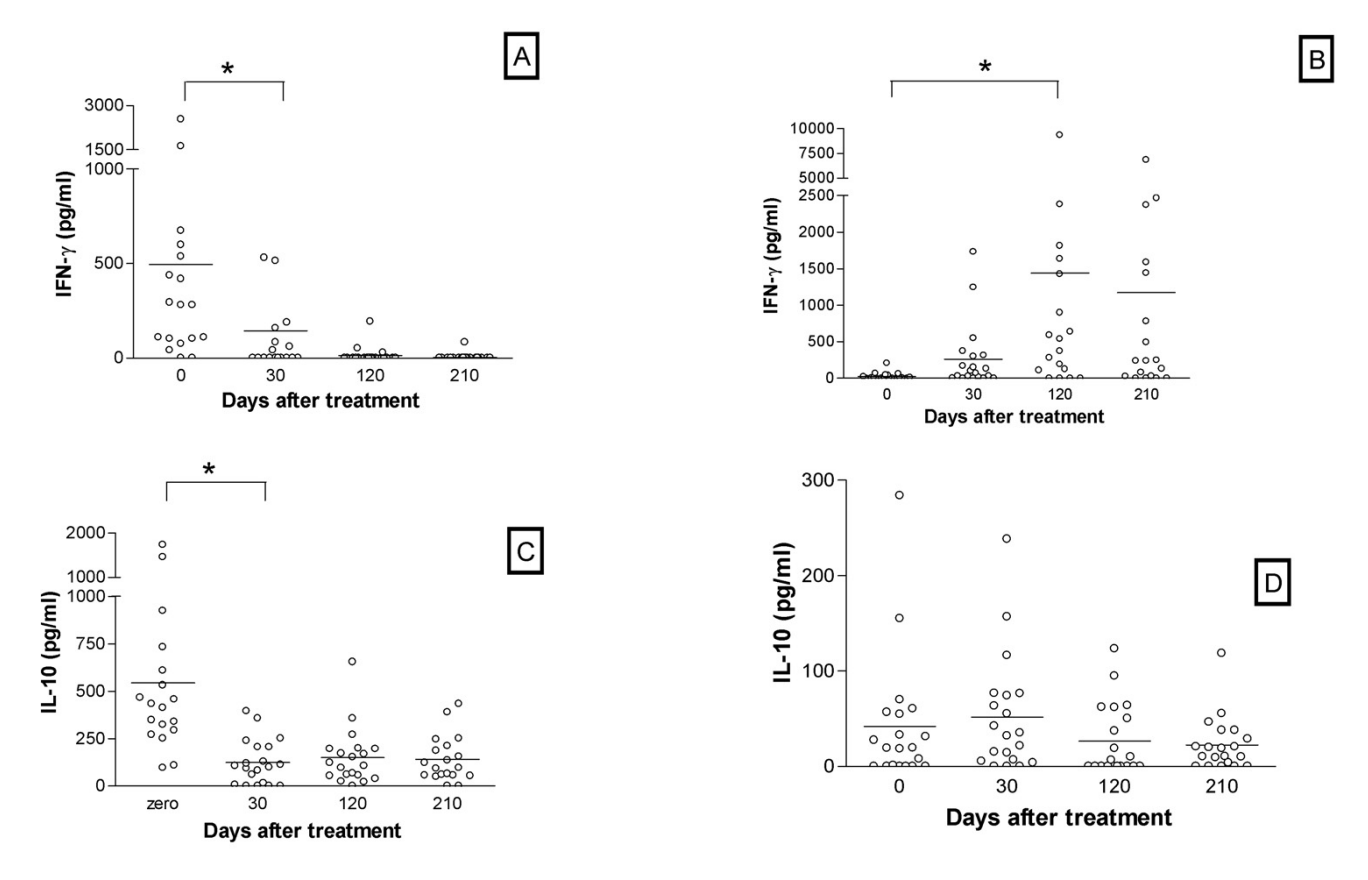
**IFN-γ and IL-10 levels in plasma and supernatant from PBMC from active VL and at different periods after treatment. **Plasma IFN-γ (A) and IL-10 (C) levels obtained by ELISA from VL patients during acute VL (day 0) and at different periods after treatment (30, 120 and 210 days) are shown. IFN-γ (B) and IL-10 (D) production obtained from PBMC from VL patients during acute VL (day 0) and at different periods after treatment (30, 120 and 210 days) restimulated *in vitro *for 72 hours with SLA (10 μg/ml) are shown.

Plasma IFN-γ levels are elevated during active disease (time 0) reaching 470.2 ± 143.4 pg/ml (n = 20) whereas normal individuals from the same endemic area exhibited levels of 15.4 ± 3.8 (n = 19). There was a significant decrease (p = 0.0012) in plasma IFN-γ levels in VL patients 30 days after treatment (155.0 ± 61.1 pg/ml), returning to the normal range 120 days post-treatment (13.7 ± 9.9; Fig. 2A). Conversely, leishmania-antigen-stimulated PBMC from VL patients do not produce IFN-γ during active disease and exhibit a significant progressive increase thereafter (p = 0.006; Fig. 2B).

Plasma levels of IL-10 were elevated during active disease (498.4 ± 97.2 pg/ml; n = 20) whereas plasma levels of IL-10 were undetectable in normal individuals from the same endemic area (n = 19). Similar to the pattern observed with IFN-γ, IL-10 circulating levels dropped dramatically 30 days after treatment (127.2 ± 125.9 pg/ml, statistically different from day zero with p < 0.001; Fig. 2C). In vitro PBMC-produced IL-10 remained low during the whole period (Fig. 2D) did not increasing in any period post-treatment.

Drop of plasma levels from 0 to 30 days of treatment was more consistent with IL-10 than IFN-γ (Fig. 2A and 2C). When evaluating antigen-driven PBMC cytokine production a different picture emerged as change in IFN-γ levels had a consistent rising time dependent pattern and IL-10 levels had a fluctuating pattern alternating rises and falls in different patients (Fig. 2B and 2D).

**Figure 3:**
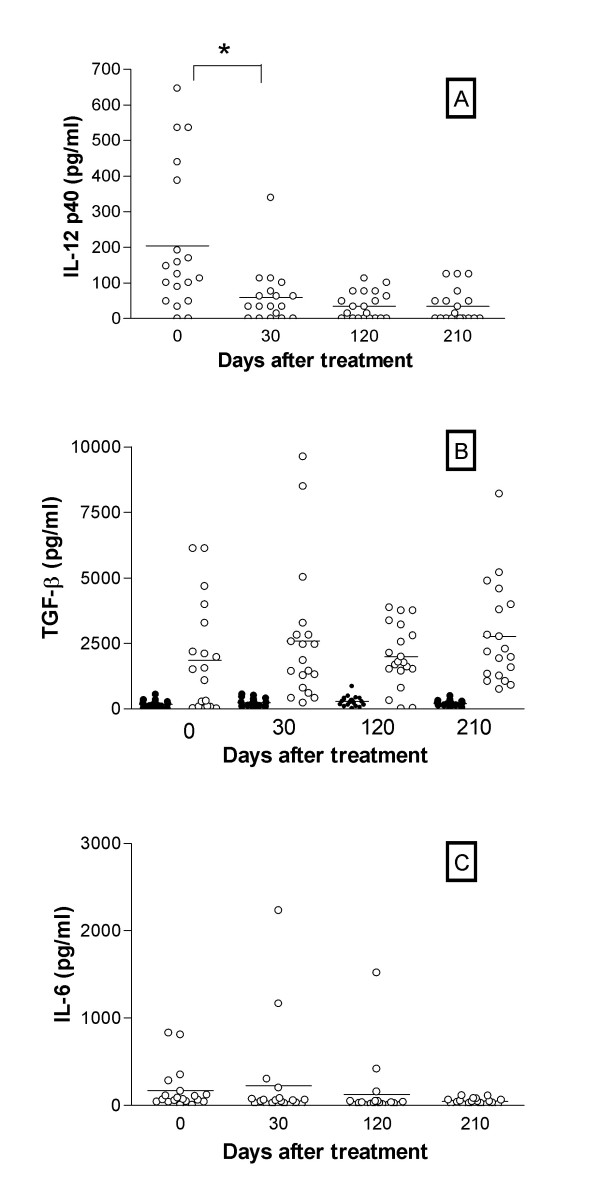
**Plasma cytokine levels from active VL and at different periods after treatment. **Plasma IL-12 p40 (A), total (open symbols) and active (closed symbols) TGF-β (B) and IL-6 (C) levels obtained by ELISA from VL patients during acute VL (day 0) and at different periods after treatment (30, 120 and 210 days) are shown in A, B, C, respectively.

IL-12 p40 plasma levels were highly elevated in five patients and moderately elevated in all other patients during active VL, with a steady declining pattern in function of time (p = 0.0007) being very low or negative in all of them by 210 days after treatment (Fig. 3A). Total, but not active, plasma TGF-β was elevated pre-treatment and remained at the same levels at all periods evaluated, and differences were not significant (Fig. 3B), proving un unreliable marker of cure. Plasma levels IL-6 were elevated during active disease in only a subgroup of patients dropping consistently over time post-treatment (p = 0.03; Fig. 3C). Interestingly, patients presenting higher levels of cytokines (IFN-γ, IL-10, Il-12 or IL-6) were not the same who showed larger spleen or liver size.

## Discussion

In the present report we studied 20 VL patients during active VL and at different time periods after treatment evaluating their cytokine and IgG levels in order to explore the time-dependent alterations of their cytokine patterns both for contributing for the comprehension of immunopathogenesis and for better defining criteria for cure. There is a marked diversity between high plasma levels of IFN-γ and the absence of its production by stimulating PBMC with leishmanial antigen during active VL. Following treatment, there is a drop in IFN-γ serum levels with concomitant rise in its production by PBMC. This might indicate that antigen-specific IFN-γ-producing cells are not circulating during active disease being released when the parasite load declines.

Our data confirm previous observations that during active human VL there is an abundant production of several cytokines [10-12,16-18]. In the present report we confirm that circulating levels of IL-12 p40 and IFN-γ itself are elevated during active VL. IFN-γ has a largely accepted role in stimulating macrophage leishmanicidal activity [19-21] and its high serum levels during active VL does not reconcile with large parasite burdens observed in this disease. IFN-γ in sera from active VL patients did not show anti-viral activity which was present in sera from tegumentary leishmaniasis or recovered VL individuals [22]. Lack of IFN-γ activity may be related to the simultaneous presence of elevated levels of IL-10, as IL-10 seems to be the main macrophage deactivating cytokine in human leishmaniasis [12,14]. We cannot rule out the role of other cytokines able to counteract IFN-γ activities such as TGF-β [23-25]. The observed fall in IL-10 levels following treatment is coincident to the control of parasite growth lending support to an important role of this cytokine in human VL.

The relative abundance of different subclasses of pathogen-specific antibody provides a good indication of the Th1/Th2 nature of systemic response in mice [26]. Determining Th1/Th2 predominance in human immune responses during leishmaniasis is not as clear [3,18,27]. The present report shows a high level of anti-Leishmania IgG, mainly comprised of IgG1 and IgG3 subclasses, with irrelevant or no increase in IgG4 or IgG2 levels in VL patients. Similar observations have been made in several parts of the World [28-32] whereas some cases from India present increased specific IgG2 levels [32,33]. We have, similarly to others, observed a modest elevation of Leishmania-specific IgG4, a subclass that has been linked to a Th2 (IL-4 or IL-13) response. However it has been difficult to detect IL-4 in sera from VL patients [10-12,34]. On the other hand, high levels of anti-Leishmania IgG1 and IgG3 subclasses and very low or absent IgG2 levels have been reported in VL [28,29,31-33] as observed in the present series. IgG2 production in man seems to depend on the IFN-γ/IL-12 influence (reviewed in [35]. IgG1 and IgG3 production although occasionally related to a Th1 human response [36], has been consistently linked in human parasitic infections to IL-10 [35]. The observed pattern of parasite-specific subclasses reinforces the notion of a major role of IL-10, leading to high IgG1 and IgG3 production, even blunting IFN-γ activity (irrelevant IgG2 production).

Elevated levels of TNF-α have been described in active VL with a marked fall after treatment [12,18,37], leading to a suggestion of its use as a criterion of cure. Patients in the present series exhibited very low plasma TNF-α levels. Early diagnosis, as in the present series, may find patients with low TNF-α levels. On the other hand, TNF-α is a very labile molecule and since we could not exclude problems in TNF-α measurement, these data are not reported. Most of the patients studied in this report were seen in outpatients' clinics, and their recovery occurred in a short period of time, probably reflecting early diagnoses. Previous reports dealt mainly with advanced in hospital VL cases and these factors might explain the differences in TNF-α levels.

A special mention should be given to DTH conversion. In the present series, no DTH conversion was observed before 120 days of treatment and even after 210 days only 40% of patients had a positive DTH. Only after a full year after effective treatment 100% of studied patients exhibited a positive DTH. Other series have reported faster DTH conversion rates [3]. It is possible that the early diagnosis and treatment as performed in our patients precluded a large expansion of antigen-reactive cells making their post-cure expansion more fastidious, whereas patients that experience a longer disease period have further expansion of their reactive cells.

Plasma levels of IL-10 show a marked decrease in most patients already 30 days after end of treatment. IL-10 levels remain detectable even after 210 days but all levels substantially lower than observed during active disease. Lymphocyte proliferation or PBMC IL-10 production in response antigen-stimulation do not exhibit a similar clear pattern as responses fluctuate among patients.

The high levels of IFN-γ observed during active VL are sharply decreased in most patients by 30 days post treatment and are almost vanished 120 days after treatment. In vitro antigen-stimulated IFN-γ production shows a specular image, being negative in all patients during active disease, positive in approximately 50% of patients by 30 days post-treatment and detectable in almost all of them by 120 days. Determining in vitro cytokine production is more cumbersome and expensive than evaluating it in plasma or serum.

## Conclusion

Plasma cytokine levels show high levels of products with largely opposed functions being difficult to determine which predominates. IFN-γ and IL-10 are the molecules most likely involved in determining fate of disease, and after treatment, there is a long delay before the immune profile returns to normal. Despite this delay, such return occurs earlier than other parameters previously proposed such as IgG levels, TNF-α circulating levels or DTH conversion. However, measuring circulating IFN-γ and/or IL-10 are not useful surrogate markers of cure in human VL as simpler clinical evaluations (absence of a palpable spleen or liver e.g.) can be used.

## Competing interests

The author(s) declare that they have no competing interests.

## Authors' contributions

AC helped on study design, data collection and interpretation of results. CF, VV, JvW participated on data collection. CB helped on study design, data collection and interpretation, JC was responsible for clinical evaluation. MB-N helped on study design, data interpretation and helped on draft manuscript. AB was responsible for the study design and manuscript elaboration.

## Pre-publication history

The pre-publication history for this paper can be accessed here:


